# Supplementary material for: Systematic exploration of local reviews of the care of maternal deaths in the UK and Ireland between 2012 and 2014: a case note review study
Source: BMJ Open. 2019 Jun 29;9(6):e029552. doi: 10.1136/bmjopen-2019-029552 (PMC6609053; doi:10.1136/bmjopen-2019-029552)
Supplement: Author's manuscript [file bmjopen-2019-029552.draft_revisions.pdf]

BMJ Open is committed to open peer review. As part of this commitment we make the peer review history of every article we publish publicly available.

When an article is published we post the peer reviewers' comments and the authors' responses online. We also post the versions of the paper that were used during peer review. These are the versions that the peer review comments apply to.

The versions of the paper that follow are the versions that were submitted during the peer review process. They are not the versions of record or the final published versions. They should not be cited or distributed as the published version of this manuscript.

BMJ Open is an open access journal and the full, final, typeset and author-corrected version of record of the manuscript is available on our site with no access controls, subscription charges or pay-per-view fees (http://bmjopen.bmj.com).

If you have any questions on BMJ Open's open peer review process please email editorial.bmjopen@bmj.com

BMJ Open

Systematic exploration of the number and quality of local reviews of the care of maternal deaths in the UK and Ireland between 2012- 2014: a case note review study

Journal:	*BMJ Open*
Manuscript ID	bmjopen-2019-029552
Article Type:	Research
Date Submitted by the Author:	31-Jan-2019
Complete List of Authors:	Cross-Sudworth, Fiona; University of Birmingham, UK, Institute of Applied Health Research<br>Knight, Marian; University of Oxford, National Perinatal Epidemiology Unit<br>Goodwin, Laura; University of Birmingham, Institute of Applied Health Research<br>Kenyon, Sara; University of Birmingham, Instititute of Applied Healthcare
Keywords:	Health & safety < HEALTH SERVICES ADMINISTRATION & MANAGEMENT, Quality in health care < HEALTH SERVICES ADMINISTRATION & MANAGEMENT, Risk management < HEALTH SERVICES ADMINISTRATION & MANAGEMENT, OBSTETRICS

SCHOLARONE™
Manuscripts

Systematic exploration of the number and quality of local reviews of the care of maternal deaths in the UK and Ireland between 2012- 2014: a case note review study

Title	Systematic exploration of the number and quality of local reviews of the care of maternal deaths in the UK and Ireland between 2012- 2014: a case note review study
Authors' names	Fiona Cross-Sudworth, Marian Knight, Laura Goodwin, Sara Kenyon
Authors' addresses, names and positions	Fiona Cross-Sudworth NIHR Intern and Research Associate; Institute of Applied Health Research, University of Birmingham, Birmingham B15 2TT, UK.<br><br>Marian Knight Professor of Maternal and Child Population Health, National Perinatal Epidemiology Unit, Nuffield Department of Population Health, University of Oxford, Oxford OX3 7LF, UK.<br><br>Laura Goodwin Research Fellow; Institute of Applied Healthcare, University of Birmingham, Birmingham B15 2TT, UK.<br><br>Sara Kenyon Professor of Evidence Based Maternity Care, Institute of Applied Healthcare, University of Birmingham, Birmingham B15 2TT, UK.
Corresponding author	Prof Sara Kenyon s.kenyon@bham.ac.uk<br>Tel: +44(0)121 414 9063

For peer review only

Abstract

Objectives – Local reviews of the care of women who die in pregnancy and post-birth should be undertaken. We investigated the quantity and quality of hospital reviews.

Design – Anonymised casenotes review.

Participants - All 233 women in the UK and Ireland who died during or up to six weeks after pregnancy from any cause related to or aggravated by pregnancy or its management in 2012-14.

Main outcome measures – The number of local reviews undertaken. Quality was assessed by the composition of the review panel, whether root causes were systematically assessed and actions detailed.

Results – The care of 177/233 (76%) women who died was reviewed locally. The care of women who died in early pregnancy and after 28 days post-birth was less likely to be reviewed as was the care of women who died outside maternity services and who died from mental health-related causes.

140 local reviews were available for assessment. Multidisciplinary review was undertaken for 65% (91/140). External involvement in review occurred in 12% (17/140) and of the family in 14% (19/140). The root causes of deaths were systematically assessed according to national guidance in 13% (18/140). In 88% (123/140) actions were recommended to improve future care, with a timeline and person responsible identified in 55% (77/140). Audit to monitor implementation of changes was recommended in 14% (19/140).

Conclusions - This systematic assessment of local reviews of care demonstrated that not all hospitals undertake a review of care of women who die during or after pregnancy and in the majority quality is lacking. The care of these women should be reviewed using a standardised robust process including root cause analysis to maximise learning and undertaken by an appropriate multidisciplinary team who are given training, support and adequate time.

Strengths and limitations of this study

- This is the first study to systematically examine the number and quality of local reviews of the care of women who died during or after pregnancy in the UK and Ireland.
- This study systematically examined the quantity and quality of local reviews of maternal deaths within the UK and Ireland over a three year period, which may not be representative of local reviews over a wider time period or in different countries.
- Each review was assessed on the basis of what was contained within the anonymised case notes provided to MBRRACE-UK, and therefore may not reflect the full procedure of review in some cases.

For peer review only - http://bmjopen.bmj.com/site/about/guidelines.xhtml

For peer review only

INTRODUCTION

For over six decades the care of all women who die during or shortly after pregnancy in the UK has been independently reviewed through a process of Confidential Enquiries (CEs). These are an internationally acknowledged method of reviewing the care of individuals who die or have severe complications in order to learn from adverse outcomes and reduce the incidence. [1 2] The principles have been utilised globally to review care of women who have died in pregnancy or in the postnatal period.[3] Countries or states that have utilised systematic confidential enquiry methodology include France, [4] Sweden,[5] Washington State, USA,[6] Tanzania,[7] Australia,[8] India,[9] and South Africa.[10] However it is less clear as to the quality of the review and it is hard to establish from the literature whether there is a standardised approach in individual countries as to the content of the review. Since 2012 a collaboration called Mothers and Babies: Reducing Risk through Audits and Confidential Enquiries across the UK (MBRRACE–UK) has been responsible for the continuation of the national programme of CEs and the surveillance of all perinatal and maternal deaths, as well as reviews of selected pre-determined significant morbidities. The CEs utilise multi-disciplinary teams of clinicians from outside the region where the woman's death occurred, to review anonymised medical records and assess the care given against national guidelines. Assessment is undertaken by these independent reviewers regarding whether care was good or improvements were noted, and if so, whether these may have made a difference to the woman's outcome. Findings from a maternal death CE published in 2018 identified that improvements in care may have made a difference to their outcome for 38 percent of all women who died. [11]

There has been some controversy over the impact of these reports. Some believe that that they have acted as a catalyst for significant improvements in maternity care across the UK and contributed towards the reduction in the national mortality rate.[12] Others, however, have questioned whether incident reporting systems such as national CE or audit are responsible for these improvements.[13] Indeed, it is argued that local review of adverse outcomes is needed, in addition to national data, in order to facilitate ownership of relevant issues and therefore increase the chance of change in practice. [14]

At local level in the UK and Ireland maternal deaths ordinarily trigger a Serious Incident review from the hospital providing the majority of care or where the woman died. Maternal deaths are rare events and usually described as 'unexpected and avoidable' and as such considered under the 'Serious Incident Framework' (SIF),[15] wherever the death. SIFs contain the explicit recommendation that contributory factors and/or root causes should be examined to identify fundamental issues and ensure a full understanding of the event to maximise the learning opportunity. The focus is to

For peer review only - http://bmjopen.bmj.com/site/about/guidelines.xhtml

consider system errors, rather than a review of individual clinicians. The National Patient Safety Agency developed a Root Cause Analysis (RCA) Investigation toolkit[16] which provides a structured way of examining nine potential contributory factors; patient, staff, task, communication, equipment, work environment, organisational, education and training and team factors. There is a currently no systematic assessment of the quality of local hospital-based reviews of the care of women who die during or shortly after pregnancy. The aim of this study was to investigate the quantity and quality of local hospital reviews following maternal deaths (during pregnancy and up until 42 days after birth) between 2012 and 2014 inclusive using MBRRACE-UK anonymised case records.

METHODS

All maternal deaths that occurred in the UK and Ireland in the three year period from 2012 to 2014 (inclusive) in early pregnancy and up to 42 days after birth were reviewed utilising anonymised case record review. Access to the anonymised case notes was via the MBRRACE-UK secure website and included case notes, statements and summaries as well as local review reports

The objectives were to identify the proportion of hospital reviews carried out when there was a maternal death relating to the time, place and cause of death; to establish which professionals were involved in reviews; if the root causes had systematically been assessed as recommended by the SIF; and whether there were resultant actions. If there were actions, whether they were individual or systematic and finally, to explore whether an audit was recommended to evaluate change in practice.

A data extraction form was developed to include key components of the SIF. For each local review three authors independently undertook the data extraction and any differences between the data were resolved between investigators.

The major causes of antenatal and postnatal deaths were examined in relation to whether local reviews of care were undertaken. These were grouped according to whether the death was related to an obstetric, medical or psychiatric cause. Obstetric deaths were those due to amniotic fluid embolism, anaesthesia, deaths in early pregnancy, haemorrhage or eclampsia and pre-eclampsia. Deaths considered to be medical in cause included cardiac deaths, those due to malignancy, neurology, sepsis, thrombosis or thromboembolism and other medical causes.

For peer review only - http://bmjopen.bmj.com/site/about/guidelines.xhtml

The quality of each review was assessed based on the SIF by examining the composition of the review panel, whether a systematic examination of the root causes (contributory factors) was undertaken and whether any actions resulted, and audit was undertaken to evidence changes to practice. The composition of each review panel was examined and the profession of those involved as documented in the review.

Patient and public involvement

This research was done without patient involvement as this study utilised anonymised case notes for secondary analysis.

RESULTS

Number and type of reviews of care of women who died

There were 262 maternal deaths that occurred between 2012 and 2014. Women who died from accidental or incidental causes such as Road Traffic Accidents (n=24) were excluded and 5 sets of case notes were unavailable which resulted in 233 maternal death cases for assessment. Of the 233 maternal deaths, 177 (76%) were reviewed by the hospital where the majority of care had been given or where the woman had died. However, there was no evidence of a review having been undertaken in 56 deaths (24%) and no evidence of change in this proportion over time.

Timing, place and cause of maternal deaths

The timing of maternal deaths was considered in relation to whether or not a review was completed. Of the 92 women who died in pregnancy, 45 (49%) of these occurred at less than 20 weeks gestation with 62% reviewed. After 20 weeks' gestation a higher proportion of the deaths were reviewed (85%) (Table 1). Of the 141 maternal deaths that occurred in the postnatal period, 78 (55%) occurred in the first week and of these 85% (n=66) were reviewed. Of the deaths between 28 and 42 days after birth, just under half (n=9, 45%) were reviewed.

Table 1: Timing (gestation and days) of women who died in pregnancy or in the early postnatal period

For peer review only - http://bmjopen.bmj.com/site/about/guidelines.xhtml

Deaths of women (gestation or days)		Reviewed	Not reviewed	TOTAL
		Number (%)	Number (%)	
Deaths in Antenatal Period (gestation)	0 - 20/40	28 (62)	17 (38)	**45**
	21 - 35/40	26 (87)	4 (13)	**30**
	36 - 42/40	14 (82)	3 (18)	**17**
**Antenatal Total**		**68 (74)**	**24 (26)**	**92**
Deaths in Postnatal period	0-6 days	66 (85)	12 (15)	**78**
	7-13 days	15 (79)	4 (21)	**19**
	14-27 days	19 (79)	5 (21)	**24**
	28-42 days	9 (45)	11 (55)	**20**
**Postnatal Total**		**109 (77)**	**32 (23)**	**141**
**TOTAL**		**177 (76%)**	**56 (24%)**	**233**

Place of death

An intensive care unit (ICU) was the most common location where women died (n=88); the care of 75% of the women who died in ICU was reviewed although maternity services were not always involved. Of the 30 women who died while being cared for in maternity services such as delivery suite, theatre, and wards, 93% (n=28) were reviewed (Table 2). Women who died in Accident and Emergency departments however, were less likely to have their cases reviewed (28/42, 67%) along with those who died in specialist units such as neurological, liver, vascular or cardiac units, or in palliative care (9/13, 69%).

Table 2: Place of death:

Place of death	Reviewed	Not reviewed	TOTAL
	Number (%)	Number (%)	
Accident & Emergency	28 (67)	14 (33)	**42**
General hospital	12 (80)	3 (20)	**15**
Home	26 (70)	11 (30)	**37**
Intensive Care Unit	66 (75)	22 (25)	**88**
Maternity services	28 (93)	2 (9)	**30**
Outdoors	8 (100)	0 (0)	**8**
Specialist units	9 (69)	4 (31)	**13**
**TOTAL**	**177 (76)**	**56 (24)**	**233**

For peer review only - http://bmjopen.bmj.com/site/about/guidelines.xhtml

Causes of Death

Of the major causes of maternal death, both obstetric and medical related deaths had higher proportions of reviews compared to deaths related to psychiatric causes (Table 3).

Table 3: Cause of death

Cause of death	Antenatal		Postnatal		TOTAL (%)
	**Reviewed** Number (%)	**Not reviewed** Number (%)	**Reviewed** Number (%)	**Not reviewed** Number (%)	
Obstetric deaths	12 (27)	4 (9)	24 (55)	4 (9)	**44** (19)
Mental health related deaths	8 (38)	4 (19)	6 (29)	3(14)	**21** (9)
Medical deaths	48 (28)	16 (10)	79 (47)	25 (15)	**168** (72)
**TOTAL**	**68 (29)**	**24 (10)**	**109 (47)**	**32 (14)**	**233** (100)

The quality of the review

Of the women who died 60% (n=140) had a documented review on the care received contained within the medical records. For a further 16% (n=37) a review was mentioned but this was not included in the medical records and so the quality could not be assessed, and 24% (n=56) had no review included in the notes. The most common type of review was entitled a Serious Incident Report (29%, n=68,), with Root Cause Analysis being the title of 18% (n=41), Hospital review of 12% (n=27) and 2% (n=4) having another title.

Composition of review panels

Sixty-five percent (91/140) of reviews were conducted by a multidisciplinary team (MDT), although this did not always include maternity services, and 18% (25/140) were conducted by a single reviewer (Table 4). Of the reviews undertaken 60% (84/140) involved obstetricians or gynaecologists and 59% (82/140) included midwives. Absence of maternity service representation was evident in cases where the death occurred at a different hospital or non-maternity department of the same hospital (for example, Accident and Emergency or Intensive Care Unit). For 16% (23/140) of reviews, the job title(s) of the professional(s) who undertook them was not documented. The family was documented as having specific questions or issues addressed by the panel in 14% (19/140) of reviews and external reviewers were involved in 12% (17/140) reviews.

For peer review only - http://bmjopen.bmj.com/site/about/guidelines.xhtml

Table 4: Professional group of reviewers

Professional group of reviewers	TOTAL n=140 (%)
Obstetrics/Gynaecology	84 (60)
Midwifery	82 (59)
Anaesthetics	41 (29)
Senior Management	48 (34)
Risk/Governance	69 (49)
Pathologist	4 (3)
External	17 (12)
Family	19 (14)
Other professional(s)	70 (50)
Not documented	23 (16)

The exact composition of the group was sometimes lacking and, while not explicitly recommended, the authors considered the optimum minimum composition of the MDT for review to be different for each of the three causes of death: Review of a maternal death from an obstetric cause should include an obstetrician, midwife and anaesthetist and yet this was only documented in 12/30 (40%) of local reviews examined. Review of a maternal death from a medical cause should include an obstetrician, midwife and specialist in whatever the medical condition, such as a cardiac specialist and yet this was documented in only 43/102 (42%) reviews examined. Review of a maternal death relating to a psychiatric cause should include an obstetrician, midwife and psychiatric specialist and yet none of those examined did (0/8). Only 55/140 (39%) of maternal deaths were considered to have been reviewed by an optimum MDT, with the composition being unclear for 71/140 (51%).

Contributory factors

Contributory factors were systematically assessed in only 13% (18/140) of local reviews using the headings listed in national guidance (see Table 5). A further 11% (15/140) used some of these factors: overall the most frequently reported factor was Communication (31/140, 22%). A small proportion of reviews (4%) utilised headings to assess care which differed to those listed in national guidance , such as Individual Knowledge and Skill, Clinical, External, Other Factors, Documentation, or Systems. In 32% (45/140) of reviews contributory factors were described in a summary paragraph only and there was no evidence that contributory factors had been examined in 36% (50/140) of local reviews examined.

For peer review only - http://bmjopen.bmj.com/site/about/guidelines.xhtml

Table 5: Inclusion of contributory factors and follow-up in RCAs:

**Root Cause Analysis content**	**Number** n=140 (%)
All individual contributory factors listed	**18** (13)
Some factors using National Patient Safety Agency headings	**15** (11)
Some factors using different headings	**5** (4)
Mixed headings	**7** (5)
Summary only	**45** (32)
No contributory factors	**50** (35)
Actions (or recommendations / learning points)	**123** (88)
No actions	**17** (12)
Systemic actions	**111** (79)
Systemic and individual actions	**12** (9)
Non-clinical actions only	**9** (6)
Timeline and person responsible identified	**77** (55)
Audit	**19** (14)

The majority of local reviews examined (88%) included actions to improve ongoing care; most of which were systemic (79%). None of the reviews reported individual actions alone, whilst 9% (12/140) included both systemic and individual actions. A small number of reviews (9/140) only included non-clinical actions such as conducting the review, completing death notifications, or supporting hospital staff. Only 14% (19/140) of all reviews of the care of women who died recommended or undertook an audit to monitor implementation of changes.

DISCUSSION

This is the first study to systematically examine the number and quality of local reviews of the care of women who died during or after pregnancy in the UK and Ireland. It shows that only three quarters of maternal deaths are reviewed and has highlighted that the care of women who died at less than 20 weeks gestation and between 28-42 days after birth was less likely to be reviewed. The care of women who died outside maternity services (for example at home) and women who died from mental health-related causes was also less likely to be reviewed. The study also suggests that a substantial proportion of these local reviews of care were not optimal, in that they were not undertaken by a multidisciplinary group, did not include root cause analysis and made relatively weak recommendations and actions.

This study systematically examined the quantity and quality of local reviews of maternal deaths within the UK and Ireland over a three year period. As such, this may not be representative of local

For peer review only - http://bmjopen.bmj.com/site/about/guidelines.xhtml

reviews over a wider time period or in different countries. Assessment was made on the basis of what was contained within the anonymised case notes provided to MBRRACE-UK, and therefore may not reflect the full procedure of review in some cases.

The study findings appears to be consistent within a wider context of reviews of care related to both maternal morbidity and perinatal death in maternity services in the UK. Shah et al[17] examined severe maternal morbidity reviews from six UK hospitals and identified that the care of some women who had severe morbidities was not reviewed and in those that were, key issues affecting the outcome were not always identified, nor were lessons evidenced as being learned. National CEs into the care of women who had term, normally formed antepartum stillbirths found that 23% had a local review carried out and only 10% were undertaken according to Royal College of Obstetrics and Gynaecology guidance.[18] While these CEs were not focused on maternal deaths, it appears that there is a lack of effective investigation of care within a hospital after a significant adverse outcome.

There is increasing evidence that the use of root cause analysis within healthcare is problematic with variable use of the investigation tools in reviews of serious incidents.[19] This is further compounded by the complexity of reviews being undertaken within tight deadlines and by a local team who may not be independent. Indeed, this may explain why such reviews commonly result in weak corrective actions and poor dissemination of findings and that repetition of similar events continues, [20 21] suggesting that lessons were not being learned and that action to address issues was inadequate. There also appears to be tension between a 'no blame' culture and a 'just culture'[22] and professional hierarchies together with the emotional tensions felt by those undertaking the review of care provide further challenges. Solutions identified include the need for professionalisation of incident investigation (including appropriate training), a need for the involvement of patient's relatives to be recognised and valued and for a better understanding of the role of blame.

The purpose of review is to learn from events and this should involve reporting, investigation, learning and action planning, implementation and closure [15]and yet of the reviews examined, not all had action plans, and just over half had a nominated person responsible for the action, with audit only recommended to check change in practice in 14%. While not systematically assessed many of the recommendations were for guidelines to be updated, training to be undertaken or dissemination of the findings. There is some evidence that easily achieved actions do not work as effectively as system changes but these are most commonly found in reviews, due to the comparatively lower financial and time costs, as well as the reduced pressure to change the culture of organisations[20]. These 'weaker' types of actions may not prevent the event from happening again.[23] Further research

For peer review only - http://bmjopen.bmj.com/site/about/guidelines.xhtml

is needed to explore the follow-up to local review including the short and long term impact of actions.

Conclusions

This study is the first to show that not all women who die in pregnancy or in the first 42 days post-birth in the UK and Ireland have their care reviewed by the local hospital. It also identified variation across hospitals concerning who was involved in reviews as well as the quality.

The care of all women who die during or after pregnancy needs to be reviewed using a standardised robust process by an appropriate multidisciplinary team. If care that can be improved is identified through the review, strong actions that will change practice are necessary with audit to monitor implementation to improve practice.

Hospitals need to allocate sufficient time for preparation, participation and appropriate follow-up for the review of care. Training is required for those involved in reviews to ensure adequate assessment of maternity service systems, culture and care, not just at the time of death, in order to come to a clear understanding of the mother's care and what if anything, could be done to prevent the same outcome happening again.

Word count: 3368 / 4000

What is already known about this topic:

- National confidential enquiries have identified that many maternal deaths are preventable
- Local hospital improvements in care are needed in order to further reduce the number of maternal deaths
- Maternal deaths should prompt a multidisciplinary review with root cause analysis within the hospital providing care with actions to address any identified issues

What this study adds:

- Systematic exploration of the reviews showed three quarters of women who died in pregnancy or within six weeks of giving birth had their care reviewed and when done rarely by an appropriate multidisciplinary team

For peer review only - http://bmjopen.bmj.com/site/about/guidelines.xhtml

- When maternal deaths were reviewed, the quality of the review could be improved suggesting that improvements in training, support and adequate time are required together with using a standardised robust process

Acknowledgments: We would like to acknowledge the contribution of the many healthcare professionals and staff from the health service and other organisations who were involved in the notification of cases, the provision of data and the assessment of individual cases in both the UK and Ireland.

Data sharing: Data are available for sharing on request from HQIP at https://www.hqip.org.uk/national-programmes/accessing-ncapop-data/#.XAFiOi10do4

Competing interests: All authors have completed the ICMJE uniform disclosure form at www.icmje.org/coi_disclosure.pdf and declare: no support from any organisation for the submitted work; no financial relationships with any organisations that might have an interest in the submitted work in the previous three years; no other relationships or activities that could appear to have influenced the submitted work.

Funding: This paper presents independent research arising from a Research Professorship award to Prof Marian Knight, NIHR-RP-011-032, supported by the National Institute for Health Research. The views expressed in this publication are those of the author(s) and not necessarily those of the NHS, the National Institute for Health Research or the Department of Health. This work was also supported by the Collaborations for Leadership in Applied Health Research and Care in the West Midlands (Grant number RGCQ17612).

Authorship: The study was designed by SK and MK. Data extraction was undertaken by FCS, LG and SK. FCS wrote the first draft of the paper which was commented on by all authors, who have approved the submitted version. All authors had access to the data in the study and can take responsibility for the integrity of the data and the accuracy of the data analysis.

Transparency declaration: The lead author affirms that this manuscript is an honest, accurate, and transparent account of the study being reported; that no important aspects of the study have been omitted; and that any discrepancies from the study as planned have been explained.

For peer review only - http://bmjopen.bmj.com/site/about/guidelines.xhtml

Ethical approval: Permission was obtained from the Healthcare Quality Improvement Partnership as data controllers to enable access to the anonymised case notes for this secondary case note review study. Research Ethics Committee approval was not required for this secondary analysis of anonymous data.

Copyright: The Corresponding Author has the right to grant on behalf of all authors and does grant on behalf of all authors, an exclusive licence on a worldwide basis to the BMJ Publishing Group Ltd to permit this article (if accepted) to be published in BMJ editions and any other BMJPGL products and sublicences such use and exploit all subsidiary rights, as set out in our licence.

For peer review only

[1] Moodley J, Pattinson RC, Fawcus S, Schoon MG, Moran N, Shweni PM on behalf of the National Committee on Confidential Enquiries into Maternal Deaths in South Africa. The confidential enquiry into maternal deaths in South Africa: a case study. 2014. http://onlinelibrary.wiley.com/doi/10.1111/1471-0528.12869/pdf Accessed 27.08.17.

[2] Knight M, Tuffnell D, Kenyon S, Shakespeare J, Gray R, Kurinczuk JJ (Eds.) on behalf of MBRRACE-UK. Saving Lives, Improving Mothers' Care - Surveillance of maternal deaths in the UK 2011-13 and lessons learned to inform maternity care from the UK and Ireland Confidential Enquiries into Maternal Deaths and Morbidity 2009-13. Oxford: National Perinatal Epidemiology Unit, University of Oxford: 2015.

[3] Lewis G. Reviewing maternal deaths to make pregnancy safer. *Best Practice & Research Clinical Obstetrics & Gynaecology.* 2008. 22(3)447-463.

[4] Agence Nationale d'Accréditation et d'Evaluation en Santé.Méthodes et Outils des démarches qualité pour les établissements de santé. 2000.

[5] Esscher A, Binder-Finnema P, Bødker B, Högberg U, Mulic-Lutvica A, Essén B. Suboptimal care and maternal mortality among foreign-born women in Sweden: Maternal audit with application of the 'migration three delays' model. BMC Pregnancy and Childbirth. 2014; 14(1).

[6] Washington State Department of Health. Maternal Mortality Review A Report on Maternal Deaths in Washington 2014–2015. 2017.

[7] Magoma M, Massinde A, Majinge C, Rumanyika R, Kihunrwa A, Gomodoka B. Maternal death reviews at Bugando hospital north-western Tanzania: a 2008–2012 retrospective analysis. BMC Pregnancy and Childbirth. 2015; 15(1):333.

[8] Australian Office of Health and Welfare. Maternal deaths in Australia 2012–2014. 2017.

[9] Gutschow K. Going 'Beyond the Numbers': Maternal Death Reviews in India. Medical Anthropology. 2016. 35(4):322-38.

[10] Moodley J PR, Fawcus S, Schoon MG, Moran N, Shweni PM. The confidential enquiry into maternal deaths in South Africa: a case study. 2014.

[11] Knight M, Bunch K, Tuffnell D, Jayakody H, Shakespeare J, Kotnis R, Kenyon S, Kurinczuk JJ (Eds.) on behalf of MBRRACE-UK. Saving Lives, Improving Mothers' Care - Lessons learned to inform maternity care from the UK and Ireland Confidential Enquiries into Maternal Deaths and Morbidity 2014-16. Oxford: National Perinatal Epidemiology Unit, University of Oxford 2018.

[12] Lewis G. Saving Mothers' Lives: the continuing benefits for maternal health from the United Kingdom (UK) confidential enquires into maternal deaths. *Seminars in Perinatology* 2012; 36:19–26.

[13] Angelow A, Black N. The use and impact of confidential enquiries in high-income countries. *BMJ Quality and Safety* 2011; 20:38e45.

[14] Stavropoulou C, Doherty C, Tosey P. How Effective Are Incident-Reporting Systems for Improving Patient Safety? A Systematic Literature Review. 2015. http://openaccess.city.ac.uk/12028/1/MQ_pre%20publication.pdf Accessed 27.8.17.

[15] NHS Improvement. Serious Incident Framework. 2015. Available at: https://improvement.nhs.uk/resources/serious-incident-framework/ Accessed 20.11.18.
[16] National Patient Safety Agency. Root Cause Analysis Investigation Tools (date unknown).
[17] Shah A, Kemp B, Sellers S, Hinton L, O'Connor M, Brocklehurst P, Kurinczuk J, Knight M. Towards optimising local reviews of severe incidents in maternity care: messages from a comparison of local and external reviews. 2016. *BMJ Quality & Safety Online* doi: 10.1136/bmjqs-2015-004960.
[18] Kenyon S, Rogers C, Johnston T on behalf of MBRRACE –UK. Follow up care and local review. In Draper ES, Kurinczuk JJ, Kenyon S. (Eds.) on behalf of MBRRACE-UK. MBRRACE-UK Perinatal Confidential Enquiry: Term, singleton, normally formed, antepartum stillbirth. Leicester: The Infant Mortality and Morbidity Studies, Department of Health Sciences, University of Leicester. 2015: p36-42.
[19] Kenyon S, Cross-Sudworth F, Keegan C, Johnston T, on behalf of MBRRACE-UK (2017). Local review of intrapartum-related death. In (Eds.) Draper ES, Kurinczuk JJ, Kenyon S on behalf of MBRRACE-UK. Perinatal Confidential Enquiry: Term, singleton, intrapartum stillbirth and intrapartum-related neonatal death. The Infant Mortality and Morbidity Studies, Department of Health Sciences, University of Leicester: Leicester. (pp.67-72).
[20] Trbovich P, Shojania KG. Root-cause analysis: swatting at mosquitoes versus draining the swamp. 2017. http://qualitysafety.bmj.com/content/26/5/350#xref-ref-4-1 Accessed 14.10.17.
[21] Kellogg KM, Hettinger Z, Shah M, Wears RL, Sellers CR, Squires M, Fairbanks RJ. Our current approach to root cause analysis: is it contributing to our failure to improve patient safety? 2017. British Medical Journal Quality and Safety; 26:381-387. Doi:10.1136/bmjqs-2016-005991.
[22] Peerally MF, Carr S, Waring J, Dixon-Woods M. The problem with root cause analysis. 2017. http://qualitysafety.bmj.com/content/26/5/417. Accessed 14.10.17.
[23] Veteran Affairs National Center for Patient Safety. RCA Tools. 2015. https://www.patientsafety.va.gov/docs/joe/rca_tools_2_15.pdf Accessed 16.11.17.

For peer review only

BMJ Open

Systematic exploration of local reviews of the care of maternal deaths in the UK and Ireland between 2012- 2014: a case note review study

Journal:	*BMJ Open*
Manuscript ID	bmjopen-2019-029552.R1
Article Type:	Research
Date Submitted by the Author:	21-May-2019
Complete List of Authors:	Cross-Sudworth, Fiona; University of Birmingham, UK, Institute of Applied Health Research<br>Knight, Marian; University of Oxford, National Perinatal Epidemiology Unit<br>Goodwin, Laura; University of Birmingham, Institute of Applied Health Research<br>Kenyon, Sara; University of Birmingham, Institute of Applied Healthcare
**Primary Subject Heading**:	Obstetrics and gynaecology
Secondary Subject Heading:	Health services research
Keywords:	Health & safety < HEALTH SERVICES ADMINISTRATION & MANAGEMENT, Quality in health care < HEALTH SERVICES ADMINISTRATION & MANAGEMENT, Risk management < HEALTH SERVICES ADMINISTRATION & MANAGEMENT, OBSTETRICS, Clinical governance < HEALTH SERVICES ADMINISTRATION & MANAGEMENT

SCHOLARONE™
Manuscripts

Systematic exploration of local reviews of the care of maternal deaths in the UK and Ireland between 2012- 2014: a case note review study

Title	Systematic exploration of local reviews of the care of maternal deaths in the UK and Ireland between 2012- 2014: a case note review study
Authors' names	Fiona Cross-Sudworth, Marian Knight, Laura Goodwin, Sara Kenyon
Authors' addresses, names and positions	Fiona Cross-Sudworth NIHR Intern and Research Associate; Institute of Applied Health Research, University of Birmingham, Birmingham B15 2TT, UK.<br><br>Marian Knight Professor of Maternal and Child Population Health, National Perinatal Epidemiology Unit, Nuffield Department of Population Health, University of Oxford, Oxford OX3 7LF, UK.<br><br>Laura Goodwin Research Fellow; Institute of Applied Healthcare, University of Birmingham, Birmingham B15 2TT, UK.<br><br>Sara Kenyon Professor of Evidence Based Maternity Care, Institute of Applied Healthcare, University of Birmingham, Birmingham B15 2TT, UK.
Corresponding author	Prof Sara Kenyon s.kenyon@bham.ac.uk<br>Tel: +44(0)121 414 9063

For peer review only

Abstract

Objectives – Local reviews of the care of women who die in pregnancy and post-birth should be undertaken. We investigated the quantity and quality of hospital reviews.

Design – Anonymised case notes review.

Participants - All 233 women in the UK and Ireland who died during or up to six weeks after pregnancy from any cause related to or aggravated by pregnancy or its management in 2012-14.

For peer review only - http://bmjopen.bmj.com/site/about/guidelines.xhtml

Main outcome measures – The number of local reviews undertaken. Quality was assessed by the composition of the review panel, whether root causes were systematically assessed and actions detailed.

Results – The care of 177/233 (76%) women who died was reviewed locally. The care of women who died in early pregnancy and after 28 days post-birth was less likely to be reviewed as was the care of women who died outside maternity services and who died from mental health-related causes.

140 local reviews were available for assessment. Multidisciplinary review was undertaken for 65% (91/140). External involvement in review occurred in 12% (17/140) and of the family in 14% (19/140). The root causes of deaths were systematically assessed according to national guidance in 13% (18/140). In 88% (123/140) actions were recommended to improve future care, with a timeline and person responsible identified in 55% (77/140). Audit to monitor implementation of changes was recommended in 14% (19/140).

Conclusions - This systematic assessment of local reviews of care demonstrated that not all hospitals undertake a review of care of women who die during or after pregnancy and in the majority quality is lacking. The care of these women should be reviewed using a standardised robust process including root cause analysis to maximise learning and undertaken by an appropriate multidisciplinary team who are given training, support and adequate time.

For peer review only

Strengths and limitations of this study

- This is the first study to systematically examine the number and quality of local reviews of the care of women who died during or after pregnancy in the UK and Ireland.
- This study systematically examined the quantity and quality of local reviews of maternal deaths within the UK and Ireland over a three year period, which may not be representative of local reviews over a wider time period or in different countries.
- Each review was assessed on the basis of what was contained within the anonymised case notes provided to MBRRACE-UK, and therefore may not reflect the full procedure of review in some cases.

INTRODUCTION

For peer review only - http://bmjopen.bmj.com/site/about/guidelines.xhtml

For over six decades the care of all women who die during or shortly after pregnancy in the UK has been independently reviewed through a process of Confidential Enquiries (CEs). These are an internationally acknowledged method of reviewing the care of individuals who die or have severe complications in order to learn from adverse outcomes and reduce the incidence. [1 2] The principles have been utilised globally to review care of women who have died in pregnancy or in the postnatal period.[3] Countries or states that have utilised systematic confidential enquiry methodology include France, [4] Sweden,[5] USA states of Washington [6] and California,[7] Tanzania,[8] Australia,[9] India,[10] and South Africa.[11] However it is less clear as to the quality of the review and it is hard to establish from the literature whether there is a standardised approach in individual countries as to the content of the review. Since 2012 a collaboration called Mothers and Babies: Reducing Risk through Audits and Confidential Enquiries across the UK (MBRRACE–UK) has been responsible for the continuation of the national programme of CEs and the surveillance of all perinatal and maternal deaths, as well as reviews of selected pre-determined significant morbidities. These CEs use multi-disciplinary teams of clinicians from outside the region where the woman's death occurred, to review anonymised case notes (medical records) and assess the care given against national guidelines. Assessment is undertaken by these independent reviewers and a consensus regarding whether care was good or improvements were noted, and if so, whether these may have made a difference to the woman's outcome is made at a multi-disciplinary meeting. Findings from a maternal death CE published in 2018 identified that improvements in care may have made a difference to their outcome for 38 percent of all women who died. [12]

There has been some controversy over the impact of these reports. Some believe that that they have acted as a catalyst for significant improvements in maternity care across the UK and contributed towards the reduction in the national mortality rate.[13] Others, however, have questioned whether incident reporting systems such as national CE or audit are responsible for these improvements.[14] Indeed, it is argued that local review of adverse outcomes is needed, in addition to national data, in order to facilitate ownership of relevant issues and therefore increase the chance of change in practice. [15]

At local level in the UK and Ireland maternal deaths ordinarily trigger a Serious Incident review from the hospital providing the majority of care or where the woman died. Maternal deaths are rare events and usually described as 'unexpected and avoidable' and as such considered under the 'Serious Incident Framework' (SIF),[16] wherever the death. SIFs contain the explicit recommendation that contributory factors and/or root causes should be examined to identify fundamental issues and ensure a full understanding of the event to maximise the learning opportunity. The focus is to

For peer review only - http://bmjopen.bmj.com/site/about/guidelines.xhtml

consider system errors, rather than a review of individual clinicians. The National Patient Safety Agency developed a Root Cause Analysis (RCA) Investigation toolkit[17] which provides a structured way of examining nine potential contributory factors; patient, staff, task, communication, equipment, work environment, organisational, education and training and team factors. There is a currently no systematic assessment of the quality of local hospital-based reviews of the care of women who die during or shortly after pregnancy. The aim of this study was to investigate the quantity and quality of local hospital reviews following maternal deaths (during pregnancy and up until 42 days after birth) between 2012 and 2014 inclusive using MBRRACE-UK anonymised case records.

METHODS

All maternal deaths that occurred in the UK and Ireland in the three year period from 2012 to 2014 (inclusive) in early pregnancy and up to 42 days after birth were reviewed utilising anonymised case record review. Access to the anonymised case notes was via the MBRRACE-UK secure website and included case notes, statements and summaries as well as local review reports

The objectives were to identify the proportion of hospital reviews carried out when there was a maternal death relating to the time, place and cause of death; to establish which professionals were involved in reviews; if the root causes had systematically been assessed as recommended by the SIF; and whether there were resultant actions. If there were actions, whether they were individual or systematic and finally, to explore whether an audit was recommended to evaluate change in practice.

A data extraction form was developed to include key components of the SIF. For each local review three authors independently undertook the data extraction and any differences between the data were resolved.

The major causes of antenatal and postnatal deaths were examined in relation to whether local reviews of care were undertaken. These were grouped according to whether the death was related to an obstetric, medical or psychiatric cause. Obstetric deaths were those due to amniotic fluid embolism, anaesthesia, deaths in early pregnancy, haemorrhage or eclampsia and pre-eclampsia. Deaths considered to be medical in cause included cardiac deaths, those due to malignancy, neurology, sepsis, thrombosis or thromboembolism and other medical causes.

For peer review only - http://bmjopen.bmj.com/site/about/guidelines.xhtml

The quality of each review was assessed based on the SIF by examining the composition of the review panel, whether a systematic examination of the root causes (contributory factors) was undertaken and whether any actions resulted, and audit was undertaken to evidence changes to practice. The composition of each review panel was examined and the profession of those involved as documented in the review.

Patient and public involvement

This research was done without patient involvement as this study utilised anonymised case notes for secondary analysis.

RESULTS

Number and type of reviews of care of women who died

There were 262 maternal deaths that occurred between 2012 and 2014. Women who died from accidental or incidental causes such as Road Traffic Accidents (n=24) were excluded and 5 sets of case notes were unavailable which resulted in 233 maternal death cases for assessment. Of the 233 maternal deaths, 177 (76%) were reviewed by the hospital where the majority of care had been given or where the woman had died. However, there was no evidence of a review having been undertaken in 56 deaths (24%) and no evidence of change in this proportion over time.

Timing, place and cause of maternal deaths

The timing of maternal deaths was considered in relation to whether or not a review was completed. Of the 92 women who died in pregnancy, 45 (49%) of these occurred at less than 20 weeks gestation with 62% reviewed. After 20 weeks' gestation a higher proportion of the deaths were reviewed (85%) (Table 1). Of the 141 maternal deaths that occurred in the postnatal period, 78 (55%) occurred in the first week and of these 85% (n=66) were reviewed. Of the deaths between 28 and 42 days after birth, just under half (n=9, 45%) were reviewed.

Table 1: Timing (gestation and days) of women who died in pregnancy or in the early postnatal period

For peer review only - http://bmjopen.bmj.com/site/about/guidelines.xhtml

Deaths of women (gestation or days)		Reviewed	Not reviewed	TOTAL
		Number (%)	Number (%)	
Deaths in Antenatal Period (gestation)	0 - 20/40	28 (62)	17 (38)	**45**
	21 - 35/40	26 (87)	4 (13)	**30**
	36 - 42/40	14 (82)	3 (18)	**17**
**Antenatal Total**		**68 (74)**	**24 (26)**	**92**
Deaths in Postnatal period	0-6 days	66 (85)	12 (15)	**78**
	7-13 days	15 (79)	4 (21)	**19**
	14-27 days	19 (79)	5 (21)	**24**
	28-42 days	9 (45)	11 (55)	**20**
**Postnatal Total**		**109 (77)**	**32 (23)**	**141**
**TOTAL**		**177 (76%)**	**56 (24%)**	**233**

Place of death

An intensive care unit (ICU) was the most common location where women died (n=88); the care of 75% of the women who died in ICU was reviewed although maternity services were not always involved. Of the 30 women who died while being cared for in maternity services such as delivery suite, theatre, and wards, 93% (n=28) were reviewed (Table 2). Women who died in Accident and Emergency departments however, were less likely to have their cases reviewed (28/42, 67%) along with those who died in specialist units such as neurological, liver, vascular or cardiac units, or in palliative care (9/13, 69%).

Table 2: Place of death:

Place of death	Reviewed	Not reviewed	TOTAL
	Number (%)	Number (%)	
Accident & Emergency	28 (67)	14 (33)	**42**
General hospital	12 (80)	3 (20)	**15**
Home	26 (70)	11 (30)	**37**
Intensive Care Unit	66 (75)	22 (25)	**88**
Maternity services	28 (93)	2 (9)	**30**
Outdoors	8 (100)	0 (0)	**8**
Specialist units	9 (69)	4 (31)	**13**
**TOTAL**	**177 (76)**	**56 (24)**	**233**

For peer review only

For peer review only - http://bmjopen.bmj.com/site/about/guidelines.xhtml

Causes of Death

Of the major causes of maternal death, both obstetric and medical related deaths had higher proportions of reviews compared to deaths related to psychiatric causes (Table 3).

Table 3: Cause of death

Cause of death	Antenatal		Postnatal		TOTAL (%)
	**Reviewed** Number (%)	**Not reviewed** Number (%)	**Reviewed** Number (%)	**Not reviewed** Number (%)	
Obstetric deaths	12 (27)	4 (9)	24 (55)	4 (9)	**44** (19)
Mental health related deaths	8 (38)	4 (19)	6 (29)	3(14)	**21** (9)
Medical deaths	48 (28)	16 (10)	79 (47)	25 (15)	**168** (72)
**TOTAL**	**68 (29)**	**24 (10)**	**109 (47)**	**32 (14)**	**233** (100)

The quality of the review

Of the women who died, 60% (n=140) had a documented local review of the care received. For a further 16% (n=37) a review was mentioned but this was not included in the case notes and so the quality could not be assessed, and 24% (n=56) had no review included in the case notes. The most common type of review was entitled a Serious Incident Report (29%, n=68), with Root Cause Analysis being the title of 18% (n=41), Hospital review of 12% (n=27) and 2% (n=4) having another title.

Composition of review panels

Sixty-five percent (91/140) of reviews were conducted by a multidisciplinary team (MDT), although this did not always include maternity services, and 18% (25/140) were conducted by a single reviewer (Table 4). Of the reviews undertaken 60% (84/140) involved obstetricians or gynaecologists and 59% (82/140) included midwives. Absence of maternity service representation was evident in cases where the death occurred at a different hospital or non-maternity department of the same hospital (for example, Accident and Emergency or Intensive Care Unit). For 16% (23/140) of reviews, the job title(s) of the professional(s) who undertook them was not documented. The family was documented as having specific questions or issues addressed by the panel in 14% (19/140) of reviews and external reviewers were involved in 12% (17/140) reviews.

For peer review only - http://bmjopen.bmj.com/site/about/guidelines.xhtml

Table 4: Professional group of reviewers

Professional group of reviewers	TOTAL n=140 (%)
Obstetrics/Gynaecology	84 (60)
Midwifery	82 (59)
Anaesthetics	41 (29)
Senior Management	48 (34)
Risk/Governance	69 (49)
Pathologist	4 (3)
External	17 (12)
Family	19 (14)
Other professional(s)	70 (50)
Not documented	23 (16)

The exact composition of the group was sometimes lacking and, while not explicitly recommended, the authors considered the optimum minimum composition of the MDT for review to be different for each of the three causes of death: Review of a maternal death from an obstetric cause should include an obstetrician, midwife and anaesthetist and yet this was only documented in 12/30 (40%) of local reviews examined. Review of a maternal death from a medical cause should include an obstetrician, midwife and specialist in whatever the medical condition, such as a cardiac specialist and yet this was documented in only 43/102 (42%) reviews examined. Review of a maternal death relating to a psychiatric cause should include an obstetrician, midwife and psychiatric specialist and yet none of those examined did (0/8). Only 55/140 (39%) of maternal deaths were considered to have been reviewed by an optimum MDT, with the composition being unclear for 71/140 (51%).

Contributory factors

Contributory factors were systematically assessed in only 13% (18/140) of local reviews using the headings listed in national guidance (see Table 5). A further 11% (15/140) used some of these factors: overall the most frequently reported factor was Communication (31/140, 22%). A small proportion of reviews (4%) utilised headings to assess care which differed to those listed in national guidance , such as Individual Knowledge and Skill, Clinical, External, Other Factors, Documentation, or Systems. In 32% (45/140) of reviews contributory factors were described in a summary paragraph only and there was no evidence that contributory factors had been examined in 36% (50/140) of local reviews examined.

For peer review only

For peer review only - http://bmjopen.bmj.com/site/about/guidelines.xhtml

Table 5: Inclusion of contributory factors and follow-up in RCAs:

**Root Cause Analysis content**	**Number** n=140 (%)
All individual contributory factors listed	**18** (13)
Some factors using National Patient Safety Agency headings	**15** (11)
Some factors using different headings	**5** (4)
Mixed headings	**7** (5)
Summary only	**45** (32)
No contributory factors	**50** (35)
Actions (or recommendations / learning points)	**123** (88)
No actions	**17** (12)
Systemic actions	**111** (79)
Systemic and individual actions	**12** (9)
Non-clinical actions only	**9** (6)
Timeline and person responsible identified	**77** (55)
Audit	**19** (14)

The majority of local reviews examined (88%) included actions to improve ongoing care; most of which were systemic (79%). None of the reviews reported individual actions alone, whilst 9% (12/140) included both systemic and individual actions. A small number of reviews (9/140) only included non-clinical actions such as conducting the review, completing death notifications, or supporting hospital staff. Only 14% (19/140) of all reviews of the care of women who died recommended or undertook an audit to monitor implementation of changes.

DISCUSSION

This is the first study to systematically examine the number and quality of local reviews of the care of women who died during or after pregnancy in the UK and Ireland. It shows that only three quarters of maternal deaths are reviewed and has highlighted that the care of women who died at less than 20 weeks gestation and between 28-42 days after birth was less likely to be reviewed. The care of women who died outside maternity services (for example at home) and women who died from mental health-related causes was also less likely to be reviewed. The study also suggests that a substantial proportion of these local reviews of care were not optimal, in that they were not undertaken by a multidisciplinary group, did not include root cause analysis and made relatively weak recommendations and actions.

This study systematically examined the quantity and quality of local reviews of maternal deaths within the UK and Ireland over a three year period. As such, this may not be representative of local

For peer review only - http://bmjopen.bmj.com/site/about/guidelines.xhtml

reviews over a wider time period or in different countries. Assessment was made on the basis of what was contained within the anonymised case notes provided to MBRRACE-UK, and therefore may not reflect the full procedure of review in some cases.

The study findings appear to be consistent within a wider context of reviews of care related to both maternal morbidity and perinatal death in maternity services. Shah et al[18] examined severe maternal morbidity reviews from six UK hospitals and identified that the care of some women who had severe morbidities was not reviewed and in those that were, key issues affecting the outcome were not always identified, nor were lessons evidenced as being learned. A comparison of American local and statewide reviews of 31 maternal deaths found that state reviews found more preventable system rather than patient factors when the cases were anonymised and investigated by an external review team. [19] National CEs into the care of women who had term, normally formed antepartum stillbirths found that 23% had a local review carried out and only 10% were undertaken according to Royal College of Obstetrics and Gynaecology guidance.[20] While some of these CEs were not focused on maternal deaths, it appears that there is a lack of effective investigation of care within a hospital after a significant adverse outcome.

There is increasing evidence that the use of root cause analysis within healthcare is problematic with variable use of the investigation tools in reviews of serious incidents.[21] This is further compounded by the complexity of reviews being undertaken within tight deadlines and by a local team who may have provided care or work alongside those who have, which may reduce objectivity. Indeed, this may explain why such reviews commonly result in weak corrective actions and poor dissemination of findings and that repetition of similar events continues [22] [23] suggesting that lessons were not being learned and that action to address issues was inadequate. There also appears to be tension between a 'no blame' culture and a 'just culture'[24] with the emotional tensions felt by those undertaking the review of care. A balance needs to be maintained between system and individual accountability; reviews should not be a scapegoat exercise while any professional failure must focus on learning and quality improvement. Suggested solutions to support quality balanced reviews include the need for professionalisation of incident investigation (including appropriate training), a need for the involvement of patient's relatives to be recognised and valued[25] and for a better understanding of the role of blame[24]. It is clear that the quality of reviews should be properly monitored and evaluated by an enhanced surveillance system, such as those not only in the UK but also elsewhere in Europe, [26] [27] North America[28] and Australasia. [29]

The purpose of review is to learn from events and this should involve reporting, investigation, learning and action planning, implementation and closure [15]and yet of the reviews examined, not all

For peer review only - http://bmjopen.bmj.com/site/about/guidelines.xhtml

had action plans, and just over half had a nominated person responsible for the action, with audit only recommended to check change in practice in 14%. While not systematically assessed many of the recommendations were for guidelines to be updated, training to be undertaken or dissemination of the findings. There is some evidence that easily achieved actions do not work as effectively as system changes but these are most commonly found in reviews, due to the comparatively lower financial and time costs, as well as the reduced pressure to change the culture of organisations[20]. These 'weaker' types of actions may not prevent the event from happening again.[30] Further research is needed to explore the follow-up to local review including the short and long term impact of actions.

Conclusions

This study is the first to show that not all women who die in pregnancy or in the first 42 days post-birth in the UK and Ireland have their care reviewed by the local hospital. It also identified variation across hospitals concerning who was involved in reviews as well as the quality.

The care of all women who die during or after pregnancy needs to be reviewed using a standardised robust process by an appropriate multidisciplinary team. If care that can be improved is identified through the review, strong actions that will change practice are necessary with audit to monitor implementation to improve practice.

Hospitals need to allocate sufficient time for preparation, participation and appropriate follow-up for the review of care. Training is required for those involved in reviews to ensure adequate assessment of maternity service systems, culture and care, not just at the time of death, in order to come to a clear understanding of the mother's care and what if anything, could be done to prevent the same outcome happening again.

Word count: 3368 / 4000

What is already known about this topic:

- National confidential enquiries have identified that many maternal deaths are preventable
- Local hospital improvements in care are needed in order to further reduce the number of maternal deaths

For peer review only - http://bmjopen.bmj.com/site/about/guidelines.xhtml

- Maternal deaths should prompt a multidisciplinary review with root cause analysis within the hospital providing care with actions to address any identified issues

What this study adds:

- Systematic exploration of the reviews showed three quarters of women who died in pregnancy or within six weeks of giving birth had their care reviewed and when done rarely by an appropriate multidisciplinary team
- When maternal deaths were reviewed, the quality of reviews could be improved suggesting that improvements in training, support and adequate time are required together with using a standardised robust process

Acknowledgments: We would like to acknowledge the contribution of the many healthcare professionals and staff from the health service and other organisations who were involved in the notification of cases, the provision of data and the assessment of individual cases in both the UK and Ireland.

Data sharing: Data are available for sharing on request from HQIP at https://www.hqip.org.uk/national-programmes/accessing-ncapop-data/#.XAFiOi10do4

Competing interests: All authors have completed the ICMJE uniform disclosure form at www.icmje.org/coi_disclosure.pdf and declare: no support from any organisation for the submitted work; no financial relationships with any organisations that might have an interest in the submitted work in the previous three years; no other relationships or activities that could appear to have influenced the submitted work.

Funding: This paper presents independent research arising from a Research Professorship award to Prof Marian Knight, NIHR-RP-011-032, supported by the National Institute for Health Research. The views expressed in this publication are those of the author(s) and not necessarily those of the NHS, the National Institute for Health Research or the Department of Health. This work was also supported by the Collaborations for Leadership in Applied Health Research and Care in the West Midlands (Grant number IS-CLA-0113-10018).

Authorship: The study was designed by SK and MK. Data extraction was undertaken by FCS, LG and SK. FCS wrote the first draft of the paper which was commented on by all authors, who have

For peer review only

For peer review only - http://bmjopen.bmj.com/site/about/guidelines.xhtml

approved the submitted version. All authors had access to the data in the study and can take responsibility for the integrity of the data and the accuracy of the data analysis.

Transparency declaration: The lead author affirms that this manuscript is an honest, accurate, and transparent account of the study being reported; that no important aspects of the study have been omitted; and that any discrepancies from the study as planned have been explained.

Ethical approval: Permission was obtained from the Healthcare Quality Improvement Partnership as data controllers to enable access to the anonymised case notes for this secondary case note review study. Research Ethics Committee approval was not required for this secondary analysis of anonymous data.

Copyright: The Corresponding Author has the right to grant on behalf of all authors and does grant on behalf of all authors, an exclusive licence on a worldwide basis to the BMJ Publishing Group Ltd to permit this article (if accepted) to be published in BMJ editions and any other BMJPGL products and sublicences such use and exploit all subsidiary rights, as set out in our licence.

---

[1] Moodley J, Pattinson RC, Fawcus S, Schoon MG, Moran N, Shweni PM on behalf of the National Committee on Confidential Enquiries into Maternal Deaths in South Africa. The confidential enquiry into maternal deaths in South Africa: a case study. 2014. Available from: http://onlinelibrary.wiley.com/doi/10.1111/1471-0528.12869/pdf Accessed 27.08.17.

[2] Knight M, Tuffnell D, Kenyon S, Shakespeare J, Gray R, Kurinczuk JJ (Eds.) on behalf of MBRRACE-UK. Saving Lives, Improving Mothers' Care - Surveillance of maternal deaths in the UK 2011-13 and lessons learned to inform maternity care from the UK and Ireland Confidential Enquiries into Maternal Deaths and Morbidity 2009-13. Oxford: National Perinatal Epidemiology Unit, University of Oxford: 2015.

[3] Lewis G. Reviewing maternal deaths to make pregnancy safer. *Best Practice & Research Clinical Obstetrics & Gynaecology.* 2008. 22(3)447-463.

[4] Agence Nationale d'Accréditation et d'Evaluation en Santé. Méthodes et Outils des démarches qualité pour les établissements de santé. 2000. Available from https://www.has-sante.fr/portail/upload/docs/application/pdf/methodes.pdf. Accessed 16.05.19.

[5] Esscher A, Binder-Finnema P, Bødker B, Högberg U, Mulic-Lutvica A, Essén B. Suboptimal care and maternal mortality among foreign-born women in Sweden: Maternal death audit with application of the 'migration three delays' model. BMC Pregnancy and Childbirth. 2014; 14(1).

[6] Washington State Department of Health. Maternal Mortality Review A Report on Maternal Deaths in Washington 2014–2015. 2017. Available from: https://www.doh.wa.gov/Portals/1/Documents/Pubs/140-154-MMRReport.pdf. Accessed 16.05.19.

[7] California Department of Public Health. The California Pregnancy-Associated Mortality Review. Report from 2002 - 2007 maternal death reviews. 2018. Available from: https://www.cdph.ca.gov/Programs/CFH/DMCAH/CDPH%20Document%20Library/PAMR/CA-PAMR-Report-1.pdf. Accessed 16.05.19.

[8] Magoma M, Massinde A, Majinge C, Rumanyika R, Kihunrwa A, Gomodoka B. Maternal death reviews at Bugando hospital north-western Tanzania: a 2008–2012 retrospective analysis. BMC Pregnancy and Childbirth. 2015; 15(1):333.

For peer review only - http://bmjopen.bmj.com/site/about/guidelines.xhtml

[9] Australian Office of Health and Welfare. Maternal deaths in Australia 2012–2014. 2017. Available from: https://www.aihw.gov.au/getmedia/a8ff4563-b8f6-43b4-826e-a861522d825a/aihw-per-92.pdf.aspx?inline=true. Accessed 16.05.19.
[10] Gutschow K. Going 'Beyond the Numbers': Maternal Death Reviews in India. Medical Anthropology. 2016. 35(4):322-38.
[11] Moodley J PR, Fawcus S, Schoon MG, Moran N, Shweni PM. The confidential enquiry into maternal deaths in South Africa: a case study. 2014. Available from: https://obgyn.onlinelibrary.wiley.com/doi/full/10.1111/1471-0528.12869. Accessed 16.05.19.
[12] Knight M, Bunch K, Tuffnell D, Jayakody H, Shakespeare J, Kotnis R, Kenyon S, Kurinczuk JJ (Eds.) on behalf of MBRRACE-UK. Saving Lives, Improving Mothers' Care - Lessons learned to inform maternity care from the UK and Ireland Confidential Enquiries into Maternal Deaths and Morbidity 2014-16. Oxford: National Perinatal Epidemiology Unit, University of Oxford 2018.

[13] Lewis G. Saving Mothers' Lives: the continuing benefits for maternal health from the United Kingdom (UK) confidential enquires into maternal deaths. *Seminars in Perinatology* 2012; 36:19–26.
[14] Angelow A, Black N. The use and impact of confidential enquiries in high-income countries. *BMJ Quality and Safety* 2011; 20:38e45.
[15] Stavropoulou C, Doherty C, Tosey P. How Effective Are Incident-Reporting Systems for Improving Patient Safety? A Systematic Literature Review. 2015. Available from: https://onlinelibrary.wiley.com/doi/full/10.1111/1468-0009.12166 Accessed 16.05.19.
[16] NHS Improvement. Serious Incident Framework. 2015. Available at: https://improvement.nhs.uk/resources/serious-incident-framework/ Accessed 20.11.18.
[17] National Patient Safety Agency. Root Cause Analysis Investigation Tools (date unknown). Available at: http://patientsafety.health.org.uk/resources/root-cause-analysis-rca-report-writing-tools-and-templates. Accessed 20.11.18.
[18] Shah A, Kemp B, Sellers S, Hinton L, O'Connor M, Brocklehurst P, Kurinczuk J, Knight M. Towards optimising local reviews of severe incidents in maternity care: messages from a comparison of local and external reviews. 2016. *BMJ Quality & Safety Online* doi: 10.1136/bmjqs-2015-004960.
[19] Geller SE, Koch AR, Martin NJ, Prentice P, Rosenberg D. *Maternal & Child Health Journal*. 19 (12)2621-2626.
[20] Kenyon S, Rogers C, Johnston T on behalf of MBRRACE –UK. Follow up care and local review. In Draper ES, Kurinczuk JJ, Kenyon S. (Eds.) on behalf of MBRRACE-UK. MBRRACE-UK Perinatal Confidential Enquiry: Term, singleton, normally formed, antepartum stillbirth. Leicester: The Infant Mortality and Morbidity Studies, Department of Health Sciences, University of Leicester. 2015: p36-42.
[21] Kenyon S, Cross-Sudworth F, Keegan C, Johnston T, on behalf of MBRRACE-UK (2017). Local review of intrapartum-related death. In (Eds.) Draper ES, Kurinczuk JJ, Kenyon S on behalf of MBRRACE-UK. Perinatal Confidential Enquiry: Term, singleton, intrapartum stillbirth and intrapartum-related neonatal death. The Infant Mortality and Morbidity Studies, Department of Health Sciences, University of Leicester: Leicester. (pp.67-72).
[22] Trbovich P, Shojania KG. Root-cause analysis: swatting at mosquitoes versus draining the swamp. 2017. http://qualitysafety.bmj.com/content/26/5/350#xref-ref-4-1 Accessed 14.10.17.
[23] Kellogg KM, Hettinger Z, Shah M, Wears RL, Sellers CR, Squires M, Fairbanks RJ. Our current approach to root cause analysis: is it contributing to our failure to improve patient safety? 2017. British Medical Journal Quality and Safety; 26:381-387. Doi:10.1136/bmjqs-2016-005991.
[24] Peerally MF, Carr S, Waring J, Dixon-Woods M. The problem with root cause analysis. 2017. http://qualitysafety.bmj.com/content/26/5/417. Accessed 14.10.17.
[25] The Royal College of Obstetricians and Gynaecologists. 2010. The Royal College of Obstetricians and Gynaecologists Green-top Guideline Number 55 *Late Intrauterine Fetal Death and Stillbirth*. Available at: https://www.rcog.org.uk/globalassets/documents/guidelines/gtg_55.pdf. Accessed 17.05.19.
[26] Donati S, Maraschini A, Lega I, D'Aloja P, Buoncristiano M, Manno V; Regional Maternal Mortality Working Group. Maternal mortality in Italy: Results and perspectives of record-linkage analysis. Acta Obstet Gynecol Scand. 2018 Nov;97(11):1317-1324. doi: 10.1111/aogs.13415. Epub 2018 Jul 20. PubMed PMID: 29956300.
[27] Deneux-Tharaux C, Saucedo M. [Enhanced system for maternal mortality surveillance in France, context and methods]. Gynecol Obstet Fertil Senol. 2017 Dec;45(12S):S3-S7. doi: 10.1016/j.gofs.2017.10.023. Epub 2017 Nov 4. French.

For peer review only - http://bmjopen.bmj.com/site/about/guidelines.xhtml

PubMed PMID: 29113880.
[28] Main EK, McCain CL, Morton CH, Holtby S, Lawton ES. Pregnancy-related mortality in California: causes, characteristics, and improvement opportunities. Obstet Gynecol. 2015 Apr;125(4):938-47. doi: 10.1097/AOG.0000000000000746. PubMed PMID: 25751214.
[29] Farquhar C, Sadler L, Masson V, Bohm G, Haslam A. Beyond the numbers: classifying contributory factors and potentially avoidable maternal deaths in New Zealand, 2006-2009. Am J Obstet Gynecol. 2011 Oct;205(4):331.e1-8. doi: 10.1016/j.ajog.2011.07.044. Epub 2011 Jul 30. PubMed PMID: 22083056.
[30] Veteran Affairs National Center for Patient Safety. RCA Tools. 2015. https://www.patientsafety.va.gov/docs/joe/rca_tools_2_15.pdf Accessed 16.05.19.

For peer review only

For peer review only - http://bmjopen.bmj.com/site/about/guidelines.xhtml
